# Successful surgical management of sudden acute obstruction of the bronchus intermedius via an interlobar approach following bilateral lung transplantation: a case report

**DOI:** 10.1186/s44215-026-00246-5

**Published:** 2026-02-23

**Authors:** Kosuke Otsubo, Chihiro Konoeda, Gouji Toyokawa, Mitsuaki Kawashima, Masaaki Sato

**Affiliations:** 1 Department of Thoracic Surgery, The University of Tokyo Hospital, 7-3-1 Hongo, Bunkyo-ku, Tokyo, 113-8655 Japan; 2 Department of Surgery and Science, Graduate School of Medical Sciences, Kyushu University, Fukuoka, Japan

**Keywords:** Lung transplantation, Airway complication, Bronchial stenosis, Bronchial sleeve resection

## Abstract

**Background:**

Lung transplantation (LTx) is an established treatment for end-stage lung disease. However, bronchial complications after LTx remain a major challenge. Bronchial stenosis is the most common bronchial complication, for which the main treatment strategies are endoscopic and surgical interventions. This report describes a case of sudden-onset bronchial stenosis following bilateral LTx that was successfully managed by surgery.

**Case presentation:**

A 46-year-old man underwent bilateral LTx from a brain-dead donor for pulmonary Langerhans cell histiocytosis and pulmonary hypertension. He was discharged 1 month later without any complications. Although there were no significant abnormal findings on chest computed tomography (CT) scans obtained 3 months after LTx, he complained of sudden-onset dyspnea at 5 months following LTx. Chest radiography showed decreased lucency in the right lower lung field, and CT demonstrated severe stenosis of the right bronchus intermedius, with the stenotic segment measuring approximately 0.8 cm in length. Limited aeration remained in the middle and lower lobes. His respiratory condition deteriorated rapidly, and he was intubated and admitted to the intensive care unit. Repeat CT performed on the following day revealed complete obstruction of the bronchus intermedius and total atelectasis of the right middle and lower lobes. After urgent transfer to our hospital, bronchoscopy revealed complete obstruction of the bronchus intermedius, indicating a need for urgent surgical intervention. Right thoracotomy was performed via the fifth intercostal space. After division of the main pulmonary artery through the interlobar fissure, sleeve resection with end-to-end reconstruction was performed for the stenotic segment of the bronchus intermedius. The postoperative course was uneventful, and the patient was discharged on postoperative day 15. No restenosis has been observed on repeat CT or bronchoscopy, and he has remained well during 2.5 years since surgery.

**Conclusions:**

Bronchial stenosis can occur after LTx even distal to the anastomotic site, particularly in the right bronchus intermedius, and may progress rapidly to complete obstruction. Surgical intervention may be an effective strategy when endoscopic management is not feasible.

## Background

Since the first lung transplantation (LTx) in 1963, advances in surgery, immunosuppressive therapy, and perioperative management have improved postoperative survival and reduced complications. However, airway complications remain a concern after LTx, with the incidence reported to be as high as 12.4% [1]. Potential risk factors include a male donor, male recipient, chronic obstructive pulmonary disease, preoperative hospitalization, early rejection, postoperative infection, perioperative extracorporeal membrane oxygenation, prolonged mechanical ventilation, telescopic anastomosis, and bilateral or right-sided LTx [1].

Airway complications post-LTx include stenosis, dehiscence, necrosis, formation of granulation tissue, malacia, and fistula; bronchial stenosis is the most common, occurring in 12%–40% of anastomotic sites and 2%–4% of distal bronchi [2]. Conventional management for airway complications generally includes bronchoscopic interventions such as balloon dilatation, stent placement, laser ablation, and brachytherapy. However, surgery is an option when these interventions are unsuccessful or not feasible. Bronchial sleeve resection with or without lung resection could be considered in selected cases of sudden acute and complete obstruction of the bronchus intermedius. In this report, we describe a case of sudden and complete obstruction of the bronchus intermedius following bilateral LTx that was managed successfully by surgical resection with bronchial reconstruction.

### Case presentation

**Fig. 1:**
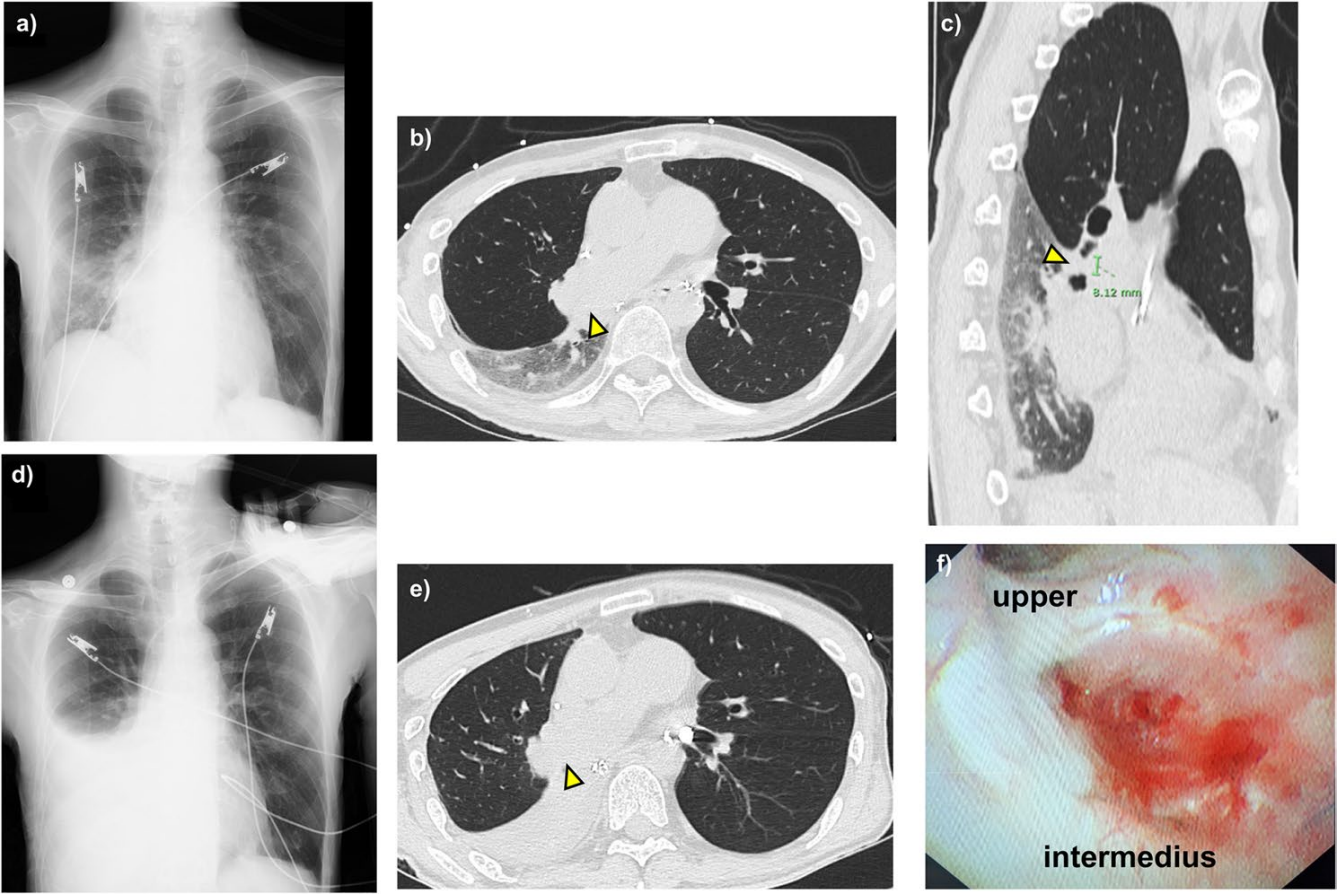
Radiological and bronchoscopic findings at the onset of respiratory deterioration 5 months after lung transplantation. **a** Chest radiography at initial presentation showing mildly decreased lucency and slight elevation of the right hemidiaphragm. **b** Chest radiography obtained after further deterioration demonstrating marked opacity of the right lower lung field. **c**,** d** Computed tomography images showing nearly complete obstruction of the bronchus intermedius (yellow arrows). The obstructed segment measured approximately 0.8 cm in length, with residual distal aeration and reduced transparency of the right middle and lower lobes. **e** Follow-up computed tomography obtained on the following day showing complete obstruction of the bronchus intermedius (yellow arrow) and total atelectasis of the middle and lower lobes. **f** Bronchoscopic findings show complete obstruction of the bronchus intermedius

A 46-year-old man with pulmonary Langerhans cell histiocytosis and pulmonary hypertension underwent bilateral LTx from a brain-dead donor. The postoperative course was uneventful, and he was discharged 1 month after surgery. Although chest CT performed at 3 months after LTx did not reveal any abnormalities, he presented with sudden-onset dyspnea 5 months after LTx. Chest radiography demonstrated decreased transparency in the right lower lung field (Fig. 1a), and CT revealed severe stenosis of the bronchus intermedius with the stenotic segment measuring approximately 0.8 cm in length; aeration of the middle and lower lobe bronchi was observed, suggesting that the stenosis/obstruction might extend distally toward the orifice of B6 and middle lobe bronchi but that both the middle and lower lobes could be preserved with at most double-barrel bronchoplasty (Fig. 1b and c). Arterial blood gas analysis at an FiO_2_ of 1.0 revealed severe hypoxemia with a PaO₂ of 46.0 mmHg and a PaCO₂ of 46.6 mmHg, indicating type II respiratory failure. The patient was emergently intubated and admitted to the intensive care unit (ICU). Chest radiography performed on the next day showed a further decrease in radiolucency in the right lower lung field (Fig. 1d). Repeat CT revealed complete occlusion of the bronchus intermedius with total atelectasis of the middle and lower lobes (Fig. 1e). He was then transferred to our institution by helicopter for emergency management. Upon arrival, arterial blood gases at an FiO₂ of 0.5 has deteriorated to a PaO₂ and PaCO₂ of 42.5 mmHg and 98.2 mmHg, respectively, indicating rapidly worsening respiratory status. No significant elevations in inflammatory markers were observed, and results for common bacterial, viral, and fungal infections were negative. Bronchoscopy revealed sudden and complete obliteration of the bronchus intermedius with the tissue appearing as a complete scar rather than fresh granulation tissue. Additionally, the sagittal CT view identified this obstruction as not being a web, but rather an obstruction nearly 1 cm thick, which precluded the option of, for example, laser recanalization followed by dilatation, prompting the decision for urgent surgical intervention (Fig. 1f).

**Fig. 2:**
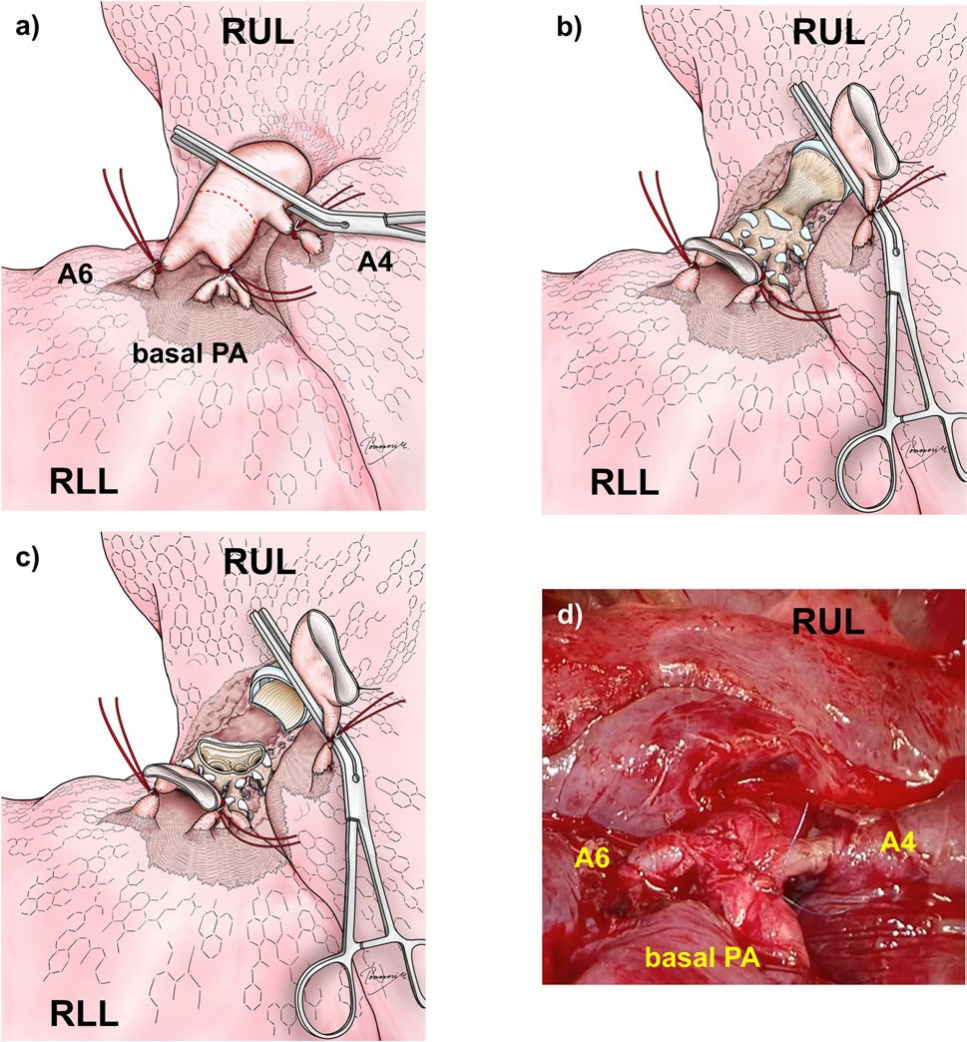
Intraoperative findings. **a** Intrapulmonary arteries (A4, A6, and the basal pulmonary artery) encircled and temporarily occluded with vessel loops and hemoclips through the interlobar fissure. After the anastomosis of the pulmonary artery, the vessel loops and hemoclips were removed, and the occlusion was released. **b** Localized stenosis of the bronchus intermedius (outlined by blue dotted line). **c** The bronchial anastomosis using end-to-end sutures with 4–0 PDS: continuous suturing for the membranous portion and 12 interrupted sutures for the cartilaginous portion. **d** Reconstruction of the divided pulmonary artery by end-to-end anastomosis with continuous 6–0 Surgipro sutures. RLL; right lower lobe; RUL, right upper lobe; PA; pulmonary artery

A right thoracotomy with sleeve resection of the bronchus intermedius and bronchial reconstruction with or without double-barrel bronchoplasty was planned. Allowing for the possibility of complex bronchoplasty, we decided to approach the bronchus intermedius through the interlobar fissure rather than via a posterior approach. The patient was positioned in the left lateral decubitus position under one-lung ventilation. A 15-cm right anterolateral thoracotomy was performed, laterally extending the previous clamshell incision. The thoracic cavity was entered through the fifth intercostal space. Severe dense pleural adhesions were found throughout the thoracic cavity and carefully dissected. The lobulation was almost complete, with minimal pleural adhesion between lobes. After exposing the interlobar pulmonary artery, the segmental pulmonary arteries (A4, A6, and basal segmental branches) were individually encircled and temporarily occluded with vessel loops and hemoclips. After administration of 5,000 units of intravenous heparin, the pulmonary artery was divided central to A^6^ (Fig. 2a). The bronchus intermedius had an hourglass-like appearance (Fig. 2b). A 23-gauge needle was inserted through the surgical field with the aid of a bronchoscope to identify the proximal resection line. By carefully examining the distal end of the resection, the orifices of B6, the basal bronchus, and the middle lobe bronchus were barely preserved (Fig. 2c), allowing for simple end-to-end anastomosis. The bronchial anastomosis was performed with 4–0 polydioxanone suture (PDS II^®^, Ethicon, Tokyo, Japan) using a continuous suture for the membranous portion and 12 interrupted sutures for the cartilaginous portion. The divided pulmonary artery was reconstructed by end-to-end anastomosis with continuous 6–0 polypropylene suture (Surgipro^®^, Medtronic, Tokyo, Japan) (Fig. 2d). Finally, we confirmed good bronchial patency, pulmonary expansion, and blood flow.

Histopathology of the resected bronchus showed erosion and ulceration of the bronchial epithelium with inflammatory cell infiltration, including neutrophils. Inflammatory granulation tissue and fibrosis at the eroded areas were found in association with luminal narrowing. Peribronchial fibrosis was also present, accompanied by arterial changes including tortuosity and calcification of the internal elastic lamina and fibrotic luminal narrowing. Partial venous recanalization is also noted. In the light of the International Society for Heart and Lung Transplantation criteria for rejection, no definite histological evidence of rejection was identified. Concomitant arterial and venous occlusive changes may have contributed to mucosal ischemia and bronchial stenosis; however, the definite cause was unclear.

**Fig. 3:**
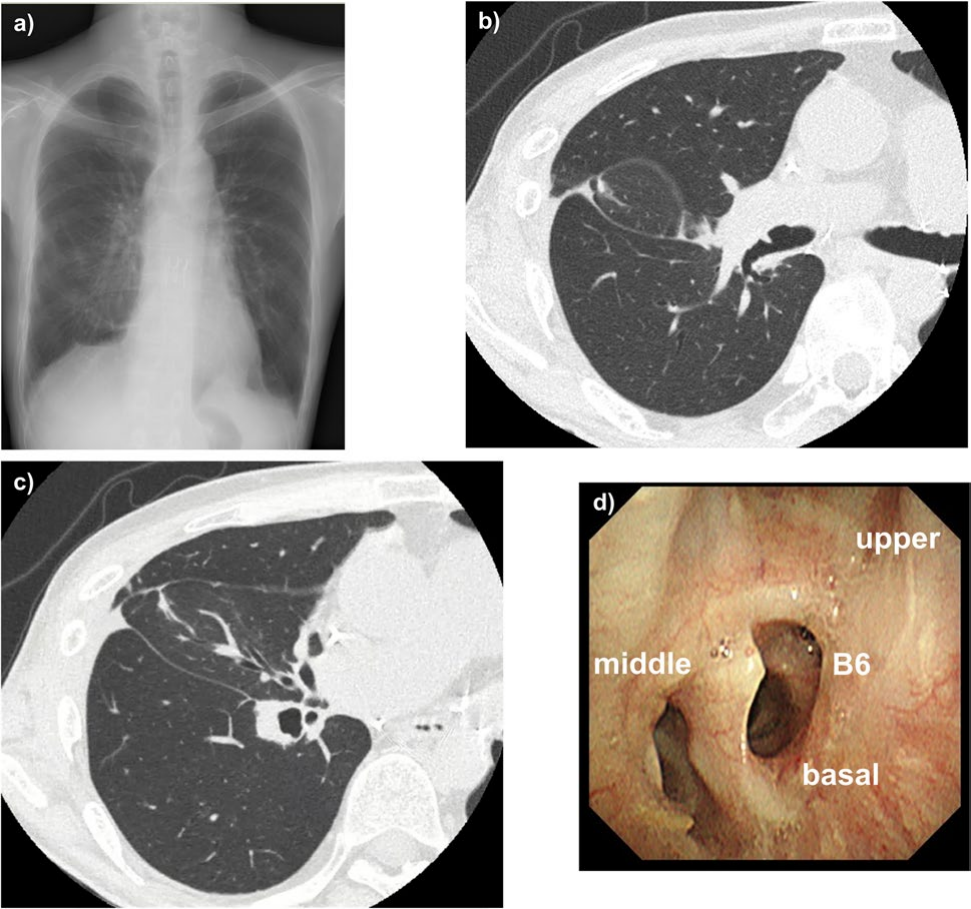
Postoperative radiological and bronchoscopic findings at 2 months after surgery. **a** Chest radiography showing improved expansion and aeration of the right lung. **b, c** Chest computed tomography images demonstrating well-aerated right middle and lower lobes with patent bronchi extending to the periphery. **d** Bronchoscopic findings showing good patency of the middle and lower lobe bronchi with no mucosal edema or restenosis

The patient remained intubated overnight and was extubated the next day. He was discharged from the ICU on postoperative day 8 and discharged home on postoperative day 15 without supplemental oxygen. Upon examination at 2 months after surgery, chest radiography showed good aeration of the right lower lung field (Fig. 3a), chest CT showed good expansion of the right middle and lower lobes with patent bronchi extending to the periphery (Fig. 3b and c), and bronchoscopy revealed good patency of the middle and lower lobe bronchi (Fig. 3d). There has been no recurrence of bronchial stenosis during 2.5 years of follow-up.

## Discussion and conclusions

This report summarizes a case of sudden obstruction of the bronchus intermedius leading to respiratory failure after bilateral LTx, which was successfully treated by sleeve resection and bronchial reconstruction without resection of any lung lobe. LTx is an established treatment for end-stage lung disease, and survival rates have improved owing to advances in immunosuppression and surgical techniques. Nonetheless, airway complications remain a serious issue, with bronchial stenosis being the most common. The reported incidence varies widely from 1.6% to 24.4% and typically occurs within 2–4 months post-transplantation [3, 4]. Bronchial stenoses can be classified as central (within 2 cm of the anastomosis) or peripheral (more than 2 cm distal to the anastomosis) [4, 5]. Peripheral stenosis is observed in approximately 3% of cases, most often involving the bronchus intermedius. In our case, the sudden acute obliteration of the bronchus intermedius developed 5 months after LTx, indicating the need for careful monitoring even during the early postoperative period.

Vanishing bronchus intermedius syndrome (VBIS), a type of airway complication following LTx, is characterized by the progressive obliteration of bronchus intermedius with chronic ischemic remodeling, often extending to distal airways, resulting in a vanishing appearance of the bronchus [6, 7]. Although the lesion in the present case was located distal to the anastomotic site and predominantly involved the bronchus intermedius—consistent with VBIS—the patient exhibited sudden acute obliteration of the bronchus intermedius. Furthermore, histopathological evaluation did not demonstrate definitive evidence of rejection according to the International Society for Heart and Lung Transplantation criteria, while concomitant arterial and venous occlusive changes were observed. Given these findings, we considered this condition to be more consistent with post-transplant distal bronchial stenosis than with VBIS or rejection, though not definitively.

Ischemic injury is considered to be the main cause of bronchial stenosis post-LTx, because the donor bronchial arteries are completely divided and not revascularized during transplantation. Therefore, the bronchial circulation depends on retrograde perfusion from the pulmonary circulation, which may be insufficient, particularly in the bronchus intermedius, which is located distal to the anastomosis, making this segment more susceptible to ischemia and subsequent fibrotic stenosis [5, 8].

Endoscopic management, such as balloon dilatation, stenting, or laser ablation, remains the preferred treatment option for bronchial stenosis post-LTx. However, in cases of complete occlusion, bronchoscopic intervention may be impossible, and surgical intervention is necessary. In the present case, bronchoscopy revealed sudden and complete obliteration of the bronchus intermedius, with no identifiable residual lumen; the tissue appeared to be a complete scar, rather than fresh granulation tissue. Moreover, the sagittal view demonstrated that this obstruction was not a web, but an almost 1 cm of thick obstruction, which was also confirmed by pathology after resection. Therefore, this condition precluded safe laser ablation and recanalization followed by passage of a guidewire or rigid bronchoscope, and surgical intervention was chosen as the initial therapeutic strategy. Faccioli et al. reported six cases of post-transplant bronchial stenosis that required surgery, and noteworthy was that all cases involved the right bronchus intermedius [9]. Although endoscopic management was initially attempted in all cases, the stenosis recurred. Three of these patients required lower sleeve bilobectomy, while one required a sleeve resection combined with middle lobectomy, indicating that post-transplant bronchial stenosis sometimes requires resection of the lower and/or middle lobes with bronchoplasty. Lobar resection was not required in two cases because the distal bronchi remained patent and the lesions could be managed adequately by sleeve resection of the bronchus intermedius or limited bronchial resection with reconstructive bronchoplasty. Our patient presented with sudden complete obstruction of the bronchus intermedius, for which endoscopic intervention was not feasible. Given the localization to the bronchus intermedius and good patency of the distal bronchi, bronchial sleeve resection without lobectomy or bilobectomy was sufficient to restore airway continuity. These findings suggest that even among patients with stenosis of the bronchus intermedius, the treatment strategy may differ depending on the mode of onset and the extent of the lesion. In selected cases, bronchial sleeve resection alone, with preservation of the lung parenchyma, may be feasible and have a good long-term outcome. The bronchus intermedius can be accessed via a posterior approach or an interlobar approach. In this case, we chose an interlobar approach with temporal division of the interlobar pulmonary artery for two reasons. First, the middle and lower lobes were considered preservable based on preoperative CT. Second, granulation tissue may have extended beyond the bronchus intermedius to B6, the basal segmental bronchus, and/or the middle lobe bronchus, which would require double-barrel or triple-barrel bronchoplasty and is somewhat complex. The interlobar approach allowed sufficient exposure of peripheral bronchial branching beyond the bronchus intermedius, allowing accurate resection and reconstruction of the bronchus. Although temporal division and reconnection of the interlobar pulmonary artery is a potential disadvantage, the procedure is likely to be feasible with minimal risk when performed by an experienced lung transplant surgeon. Another issue with this approach could be incomplete lobulation, which necessitates extra maneuvers to reach the interlobar pulmonary artery and may increase the risk of prolonged air leakage as a result of lung parenchymal injury, although lobulation was well developed in our patient.

This case highlights the importance of timely and appropriate intervention for stenosis of the bronchus intermedius. Prompt surgery is sometimes necessary in patients who experience rapid and complete obstruction of the bronchus intermedius.

## Data Availability

Not applicable.

## Abbreviations

CT: computed tomography
ICU: intensive care unit
LTx: lung transplantation
PaCO₂: arterial partial pressure of carbon dioxide
PaO₂: arterial partial pressure of oxygen
FiO₂: fraction of inspired oxygen
VBIS: vanishing bronchus intermedius syndrome
